# ATACdb: a comprehensive human chromatin accessibility database

**DOI:** 10.1093/nar/gkaa943

**Published:** 2020-10-30

**Authors:** Fan Wang, Xuefeng Bai, Yuezhu Wang, Yong Jiang, Bo Ai, Yong Zhang, Yuejuan Liu, Mingcong Xu, Qiuyu Wang, Xiaole Han, Qi Pan, Yanyu Li, Xuecang Li, Jian Zhang, Jun Zhao, Guorui Zhang, Chenchen Feng, Jiang Zhu, Chunquan Li

**Affiliations:** School of Medical Informatics, Daqing Campus, Harbin Medical University, Daqing 163319, China; School of Medical Informatics, Daqing Campus, Harbin Medical University, Daqing 163319, China; School of Medical Informatics, Daqing Campus, Harbin Medical University, Daqing 163319, China; School of Medical Informatics, Daqing Campus, Harbin Medical University, Daqing 163319, China; School of Medical Informatics, Daqing Campus, Harbin Medical University, Daqing 163319, China; School of Physics and Electronic Engineering, Northeast Petroleum University, Daqing 163318, China; School of Medical Informatics, Daqing Campus, Harbin Medical University, Daqing 163319, China; School of Medical Informatics, Daqing Campus, Harbin Medical University, Daqing 163319, China; School of Medical Informatics, Daqing Campus, Harbin Medical University, Daqing 163319, China; School of Medical Informatics, Daqing Campus, Harbin Medical University, Daqing 163319, China; School of Medical Informatics, Daqing Campus, Harbin Medical University, Daqing 163319, China; School of Medical Informatics, Daqing Campus, Harbin Medical University, Daqing 163319, China; School of Medical Informatics, Daqing Campus, Harbin Medical University, Daqing 163319, China; School of Medical Informatics, Daqing Campus, Harbin Medical University, Daqing 163319, China; School of Medical Informatics, Daqing Campus, Harbin Medical University, Daqing 163319, China; School of Medical Informatics, Daqing Campus, Harbin Medical University, Daqing 163319, China; School of Medical Informatics, Daqing Campus, Harbin Medical University, Daqing 163319, China; School of Medical Informatics, Daqing Campus, Harbin Medical University, Daqing 163319, China; School of Medical Informatics, Daqing Campus, Harbin Medical University, Daqing 163319, China

## Abstract

Accessible chromatin is a highly informative structural feature for identifying regulatory elements, which provides a large amount of information about transcriptional activity and gene regulatory mechanisms. Human ATAC-seq datasets are accumulating rapidly, prompting an urgent need to comprehensively collect and effectively process these data. We developed a comprehensive human chromatin accessibility database (ATACdb, http://www.licpathway.net/ATACdb), with the aim of providing a large amount of publicly available resources on human chromatin accessibility data, and to annotate and illustrate potential roles in a tissue/cell type-specific manner. The current version of ATACdb documented a total of 52 078 883 regions from over 1400 ATAC-seq samples. These samples have been manually curated from over 2200 chromatin accessibility samples from NCBI GEO/SRA. To make these datasets more accessible to the research community, ATACdb provides a quality assurance process including four quality control (QC) metrics. ATACdb provides detailed (epi)genetic annotations in chromatin accessibility regions, including super-enhancers, typical enhancers, transcription factors (TFs), common single-nucleotide polymorphisms (SNPs), risk SNPs, eQTLs, LD SNPs, methylations, chromatin interactions and TADs. Especially, ATACdb provides accurate inference of TF footprints within chromatin accessibility regions. ATACdb is a powerful platform that provides the most comprehensive accessible chromatin data, QC, TF footprint and various other annotations.

## INTRODUCTION

Genome-wide identification of chromatin accessibility is important for detecting regulatory elements and understanding transcriptional regulation governing biological processes such as cell fate determination, cell differentiation and diseases development (1,2). In cancer cells, chromatin accessibility profiling has been proven to be used to identify transcription factor binding sites (TFBSs) and predict regulatory networks for studying transcriptional regulation mechanisms (3). In the human retinae, chromatin accessibility-associated transcription factors (TFs), as critical regulators for photoreceptor differentiation, played important roles in photoreceptor maturation at the late stage of retinae development (4). In T-cell lymphoma, changes in chromatin accessibility were correlated with gene expression of IFNG, resulting in distinct chromatin responses in leukemic and host CD4+T cells (5). Lugena *et al.* detected significant TF footprints within accessible chromatin regions in brains of wild-type monarchs, which revealed the rhythmic genes and regulation modes in the monarch brain (6). Disease-associated sequence variations are enriched in chromatin accessibility regions (7). For example, Type 2 diabetes-associated single-nucleotide polymorphisms (SNPs) within chromatin accessibility regions in human islets, contributed to islet dysfunction and failure (8). In the brain tissue, the SNP heritability of schizophrenia enriched in accessible chromatin regions contributes to the risk of schizophrenia (9). In colorectal cancer, loss of ARID1A located at enhancers leads to dramatic changes in chromatin accessibility, and influences the expression of MET in colorectal cancer cell growth and adhesion (10). Many studies have revealed that DNA methylation has a complex interplay with accessible chromatin. For example, Rizzardi *et al.* found that neuronal brain region-specific DNA methylation within chromatin accessibility regions mediated neuropsychiatric trait heritability (11). Together, these studies confirmed the significance of chromatin accessibility in addressing key issues associated with biological processes, cell differentiation, cancer biology and disease development.

In recent years, there have been several high-throughput methods to profile chromatin accessibility, such as ATAC-seq (12), DNase-seq (13), FAIRE-seq (14) and MNase-seq (15). Compared to other technologies, ATAC-seq is a powerful technology with high accuracy and sensitivity to profile genome-wide chromatin accessibility (12,16,17). Although several relevant publicly resources such as Cistrome (18), TCGA (19) and ENCODE (20) store some chromatin accessibility data, there is no chromatin accessibility database based on ATAC-seq that focuses on collecting a large number of human ATAC-seq chromatin accessibility regions, or that provides the comprehensive detailed information about standardized curation, quality control (QC), TF footprints and various other annotation information. In addition, several databases store chromatin accessibility data based on DNase-seq datasets, including GTRD (21), EpiRegio (22), DeepBlue (23) and OCHROdb (24). However, GTRD, EpiRegio and DeepBlue are focused on gene regulation for ChIP-seq and DNase-seq data, and only supported some chromatin accessibility data. OCHROdb is a database based on chromatin accessibility data, it only supports DNase-I samples. Human ATAC-seq datasets are accumulating rapidly, which promotes an urgent need to comprehensively collect and effectively process these data. More importantly, quality measure processes are necessary for ATAC-seq experiment. Assessing the quality of ATAC-seq is used to help researchers reach more precise assumptions or conclusions (25). Footprints reveal the presence of DNA-binding proteins at each site in the accessible region, which promotes a better understanding of gene regulation and chromatin dynamics (12). Together, building a valuable resource to integrate, annotate and analyze these human chromatin accessibility data can help researchers understand epigenomic mechanisms deeply, and discover more biological functions in accessible chromatin regions.

In the present study, we developed a comprehensive chromatin accessibility database for human (ATACdb, http://www.licpathway.net/ATACdb), which provides a large number of human chromatin accessibility data based on ATAC-seq. ATACdb contains 52 078 883 regions from 1493 ATAC-seq samples, which were manually curated from over 2200 chromatin accessibility samples associated with ATAC-seq data from NCBI GEO/SRA (26,27). Various detailed (epi)genetic annotation information about chromatin accessibility regions are supported in our database. ATACdb can display a QC report for each sample, including mean insert size and standard deviation, TSS enrichment score and Fraction of Reads in Peaks (FRiP). To view a QC report intuitively, ATACdb displays diagnostic plots for samples. The database further supports TF footprint analysis for inferring TFBS and provides exhaustive information for footprint. ATACdb is a user-friendly database to query, browse and visualize information associated with chromatin accessibility regions.

## MATERIALS AND METHODS

### Data collection and identification of accessible chromatin regions

**Figure 1.**
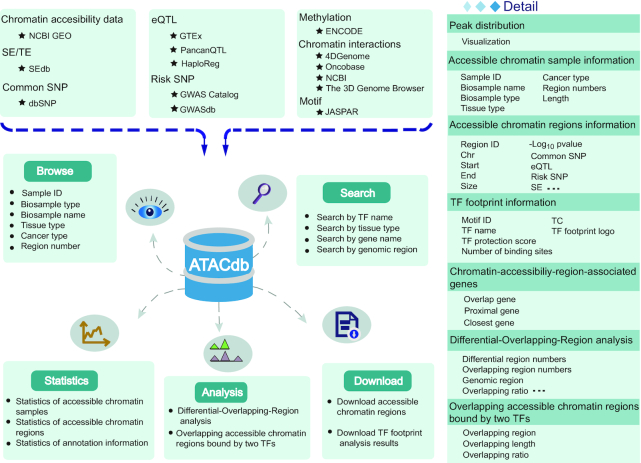
Database content and construction. Chromatin accessibility regions in ATACdb were calculated based on human ATAC-seq data. Genetic and epigenetic annotations were collected or calculated, including super-enhancers, typical enhancers, TFs, common SNPs, risk SNPs, eQTLs, LD SNPs, DNA methylation sites, 3D chromatin interactions and TADs. Users can determine the scope of the chromatin accessibility data query through four paths: genomic region-based query, tissue-category-based query, TF-based query and gene-based query. ATACdb contains analytical tools and multiple functions to browse, search, download and visualize chromatin accessibility information.

In ATACdb, we manually collected over 2200 publicly available human ATAC-seq samples. Notably, we first integrated all sample identifiers (GSM ID) from GEO (26) using the keyword of ‘human species[Organism]’ and ‘ATAC-seq’. All chromatin accessibility samples were manually curated from NCBI GEO/SRA (26,27) (Figure 1). To attain more accuracy, all samples were examined in the GEO sample description text and non-compliant samples were filtered out, such as single-cell ATAC-seq. Second, for sequencing data, we integrated Trim Galore (v1.18) (http://www.bioinformatics.babraham.ac.uk/projects/trim_galore/) for trimming of the adapter and low quality reads. This step avoided unqualified sequences that affected the alignment results. Third, we used Bowtie2 (v2.25) (28) for aligning reads to the human reference genome (hg19) that was downloaded from UCSC Genome Bioinformatics with the following parameters (-X 2000 –no-mixed –no-discordant). Fourth, the produced SAM file by Bowtie2 (v2.25) (28) was used by the SAMtools (v1.90) (29) and Picard (http://broadinstitute.github.io/picard/) for viewing and processing. SAMtools was used to index the resulting alignments in the SAM/BAM format and Picard was used to remove duplicate nucleotide sequences. Finally, MACS2 (v2.1.2) (30) was used to identify accessible chromatin regions, as well as the summit of each ATAC-seq peak with the following parameters ‘–broad–SPMR –nomodel –extsize 200 -q 0.01’. The ENCODE blacklisted regions (20,31) often had extremely high read coverage, and thus were discarded in ATACdb (32).

### ATAC-seq quality control

The QC measurement is an important feature of ATAC-seq datasets. We provided four different QC metrics of ATAC-seq samples, including mean insert size and corresponding standard deviation of paired-end libraries (12) using Picard (http://broadinstitute.github.io/picard/), TSS enrichment score and FRiP using the ENCODE consortium (33,34). We preferred the mean insert size as a superior metric of quality assessment, because it was estimated after trimming off the outliers in from the original insert-size distribution. The TSS enrichment score indicated the average depth of the TSS of genes and the FRiP indicated fraction of mapped reads falling into the peak regions. In order to view QC measures intuitively for users, we displayed a graph showing the insert size distribution in the sample detail page. The spatial frequency of chromatin-dependent periodicity coincides with nucleosome (12). We displayed a histogram of the insert size distribution, which reflected decreasing and periodical peaks corresponding to the nucleosome free regions (nfr) (<100 bp), mononucleosomes (∼200 bp), dinucleosomes (∼400 bp) and trinucleosomes (∼600 bp), to test ATAC-seq experiment (12,25,35). The high-quality ATAC-seq experiment could produce valuable information about improving the preparation of samples (Supplementary Figure S1A). On the contrary, the typical insert size distribution plot for a failed ATAC-seq experiment is shown in Supplementary Figure S1B. Low_quality ATAC-seq experiments might have resulted from a high ratio of Tn5 transposase or biased size selection during library preparation (21). Based on the overall QC distributions, we established the thresholds of QC characteristics and filtered out a few low quality samples. Overall, these steps identified 52 078 883 accessible chromatin regions from 1493 ATAC-seq samples.

### TF footprint analysis

TF footprint analysis can significantly improve the accuracy of TFBS identification, which has unique ability to assess changes in the activity of TFs and discover cell-specific TFBS (12). ATAC-seq-based genomic footprint refers to the pattern where an active TF binds to DNA and prevents Tn5 transposase cleavage within the binding site, which is a fast growing area of chromatin accessibility study (36,37). More importantly, TF footprint analysis has been used to detect TF occupancy, the effects of genetic variants in TF binding, and to identify cell- and lineage-selective transcriptional regulators (38–40). To explore more biological functions of TF footprints, ATACdb predicts TFBS with footprints using HINT (41), which is based on hidden Markov models. By incorporating all these biases with the parameters: ‘-bc’, HINT can predict TF footprints, and significantly surpasses other competing methods (36). Motifs from JASPAR were used to do motif matching for footprints (42).

Finally, all motif predicted binding sites were calculated by matching all position weight matrices against the human reference genome in ATACdb. TFs with the Tag Count (TC), protection score, number of binding sites and footprint logo were identified for each sample. We used TC to rank footprint predictions, which indicated the number of reads around putative TFBSs (25). To further understand the footprint, we provided the protection score to discover footprints with potential short residence binding times (43). The protection score was calculated by measuring the different Tn5 digestion numbers between TFBS and flanking regions (36,37). The profiles for each motif, which can indicate the activity of TF intuitively, were displayed in ATACdb. We have filtered out TFs with ≤10 binding sites. We have now added some new ‘Threshold’ options, including ‘Protection score threshold’, ‘TC threshold’ and ‘Number of binding sites threshold’, which allows users to set different thresholds to ensure TFs are high-activity and cell-type-specific in our website. For example, we set a default threshold of the number of binding sites (the default value: 100). All TF footprints for each sample can be downloaded in the ‘Download’ page.

### Chromatin accessibility region annotation

Accessible chromatin region annotation can promote the investigations in biological processes and diseases. ATACdb provides detailed (epi)genetic annotation information in accessible chromatin regions, including TFs, super-enhancers, typical enhancers, common SNPs, risk SNPs, eQTLs, LD SNPs, DNA methylation sites 3D chromatin interactions and TADs. We used BEDTools (v2.25.0) (44) to annotate corresponding information in accessible chromatin regions, and displayed details of the annotation using interactive tables.

#### Transcription factors (TFs)

ATACdb provides two types of analysis methods for detecting TFs binding to the accessible chromatin region. One is the TF footprint (discussed in the above section). Another is a sequence-based prediction for motif frequency (motif scan). For motif scan analysis, we used the FIMO (45) tool from the MEME (46) suite to predict putative TFBSs from sequences within accessible chromatin regions. The motif information were obtained from the JASPAR database (42). We have scanned for occurrences of motifs in every accessible chromatin region for each ATAC sample. And we have identified individual candidate binding sites or protein motifs in a total of 52 078 883 accessible chromatin regions in ATACdb. We found that some motifs are short. They may not be found if users set a too stringent *P*-value of FIMO. Therefore, we identified DNA-binding sequence motifs with a *P*-value threshold of 1e−4, make sure that short motifs were also well represented in our database. We further added some ‘FIMO threshold’ options allowing users to select different parameters. This annotation can help users systematically investigate patterns of TF bindings within accessible chromatin regions, which is of great significance for further understanding gene regulation and biological regulatory networks.

#### Super-enhancers/typical enhancers

The complex relationship between chromatin accessibility and super-enhancers may help decipher transcriptional activity and gene expression mechanisms (41). To annotate the potential roles of super-enhancers and typical enhancers within accessible chromatin regions, we collected a total of 331 146 super-enhancers and 6 629 274 typical enhancers from SEdb (47). We annotated super-enhancers and typical enhancers to accessible chromatin regions, and the detailed information were provided, including sample name, ChIP density, rank and associated genes in the closest strategy (47–49).

#### Common SNPs/eQTLs/risk SNPs/LD SNPs

To annotate the effects of SNPs located in accessible chromatin regions, we obtained 38 063 729 common SNPs from dbSNP (50) and filtered out SNPs with a minimum allele frequency (MAF) < 0.01. We obtained mutation data and phased genotype data from the 1000 Genomes Project phase 3 (51) and separated out mutations with MAF > 0.05 using VCFTools (v0.1.13) (52). Plink (v1.9) (53) was used to calculate the LD SNPs (*r*2 = 0.8) of five super-populations (African, Ad Mixed American, East Asian, European and South Asian). For risk SNP, a total of 264 514 risk SNPs were obtained from the GWAS Catalog (54) and GWASdbv2.0 (55). The functional annotations for SNPs and insertion/deletions variants in the human disease/traits were also collected. We obtained 2 886 133 human eQTLs and 31 080 511 eQTL-gene pairs from PancanQTL (56), HaploReg (57) and GTEx v5.0 (58).

#### Methylations/chromatin interactions/TADs

The functional interplay between chromatin accessibility and methylation provides information about the DNA sequence and TF binding at methylation sites, which is significant for the genome-wide study of gene regulation (59). For better understanding of the relationships between methylation and accessibility, we obtained 30 392 523 methylation sites of 450k array from ENCODE (31). Chromatin interaction data can help users understand gene expression mechanisms. We obtained chromatin interaction data, including Hi-C, ChIA-PET, 3C, 4C and 5C. Ultimately, 29 920 872 interactions were collected from Oncobase (60), 4DGenome (61), NCBI (26) and the 3D Genome Browser (62).

The complex relationship between chromatin accessibility region and TAD play an important role in regulation of gene expression. To better understand chromatin accessibility regions and their associated genes within TADs, we collected TADs covering 21 tissue types from the 3D Genome Browser (62). We provided TAD annotation information for chromatin accessibility regions and related details.

### Chromatin-accessibility-region-associated genes

We analyzed accessible chromatin regions and determined their associated genes, which accelerated the characterization of gene regulation and biological processes. We used a python script from ROSE (ROSE_geneMapper.py) (63) to predict chromatin-accessibiliy-region-associated genes. Notably, we calculated the distance of each peak to the ±1 kb region around the TSS and annotated the peak to the corresponding genes. Chromatin-accessibiliy-region-associated genes were identified by ROSE_geneMapper on the basis of closest, overlap and proximal strategies (47–49,63). All associated genes identified from three strategies were provided in ATACdb, which could be used as a gene-based query method in ATACdb.

### Peak annotation visualization

ATACdb implements visualization functions of peak annotation using ChIPseeker (64). We supported visualization of ATAC-seq peaks in different ways, including with displays of peak coverage over chromosomes and profiles of peaks binding to the TSS region. For each sample, we exhibited pie charts of annotated genomic features using the *annotatePeak* function (64), which can report the proportion of genomic region annotations (promoter, 5′ UTR, 3′ UTR, exon, intron, downstream and intergenic). The *peakHeatmap* function (64) was used to visualize profiles of ATAC peaks binding to the TSS region. ATACdb exhibits heatmaps of peaks binding to the TSS region (±1 kb) for each sample, which makes it easier for users to compare among different ATAC-seq experiments.

## DATABASE USE AND ACCESS

### A search interface for retrieving chromatin accessibility data

**Figure 2.**
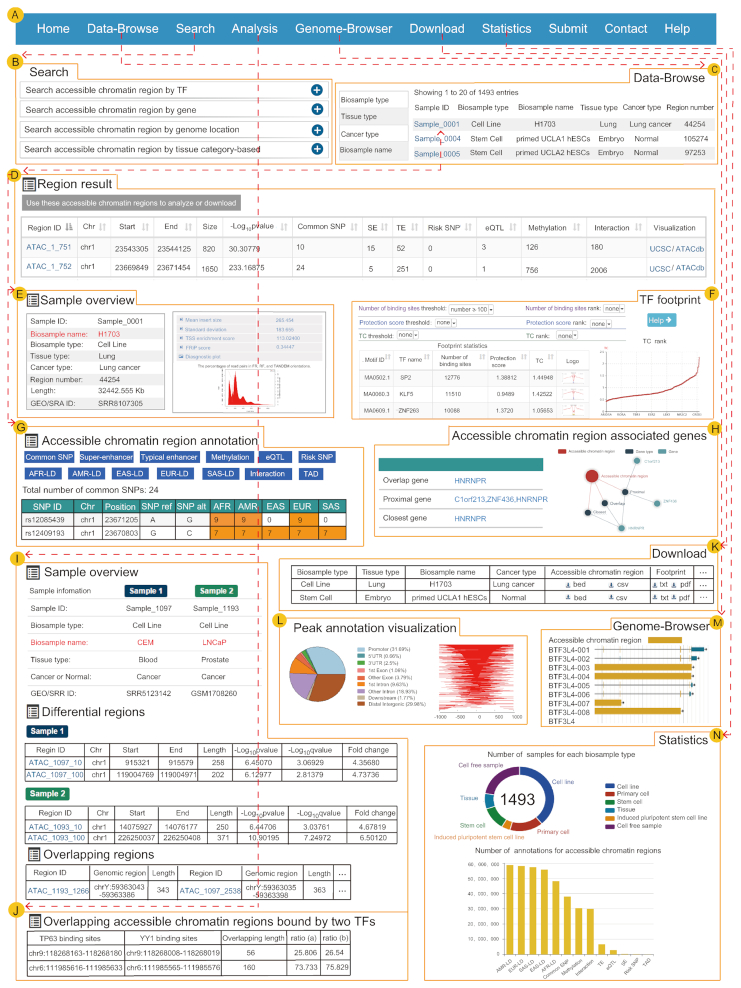
The main functions and usages of ATACdb. (**A**) The navigation bar of functions in ATACdb. (**B**) Users can query chromatin accessibility regions through four paths: ‘Search by genomic region’, ‘Search by tissue type’, ‘Search by TF name’ and ‘Search by gene name’. (**C**) Browse samples. (**D**) Table of search results including region ID, chr, start, end, size, -log_10_*P* value, common SNPs, super-enhancers, typical enhancers, risk SNPs, eQTLs, DNA methylation sites, 3D chromatin interactions and visualization (genome browser). (**E**) Sample information including biosample name, biosample type, tissue type, cancer type, region number, length, GEO/SRA ID and QC report. (**F**) The detailed information of TF footprint. (**G**) The detailed interactive table of annotation information. (**H**) Accessible chromatin regions associated genes are identified through three strategies. Network diagram about these regions is displayed. (**I**) Analysis of differential and overlapping accessible chromatin regions between two samples. (**J**) Analysis of overlapping accessible chromatin regions bound by two TFs. (**K**) Data download. (**L**) Visualization of peak annotation. (**M**) Genome browser. (**N**) Sample and annotation statistics in ATACdb.

ATACdb is a powerful platform with user-friendly search options to retrieve chromatin accessibility data (Figure 2A and B). Users can determine the scope of chromatin accessibility data query through four paths, including ‘Search by genomic region’ (input genomic position), ‘Search by tissue type’ (input tissue name of interest), ‘Search by TF’ (input TF name of interest) and ‘Search by gene’ (input gene name and identification strategies). In the genomic region-based query, users can input genomic position, and ATACdb will identify accessible chromatin regions overlapping with the submitted region. Based on the TF query, users can obtain all accessible chromatin regions bound by the TF through submiting a TF of interest. Users may also submit a gene name, and accessible chromatin regions associated with it can be returned via relationships between the accessible chromatin regions and associated genes, which are identified in three strategies including closest, overlap and proximal (47–49). In the tissue-based query, users can select ‘Tissue type’ and ‘Biosample type’ for customizing filters. ATACdb can display accessible chromatin regions associated with a specific type of tissue on the result page.

The brief information on the search results is displayed in a table on the result page. The table describes region ID, genome location, length, fold change, -log_10_*P*/log_10_*q* value and detailed (epi)genetic information in accessible chromatin regions (Figure 2D). The result page provides the QC report of ATAC-seq data including four measure scores and a histogram (Figure 2E). Users can view accessible chromatin region distribution in chromosomes. For each sample, ATACdb enables TF footprint analysis results, including TFs with the TC, TF protection score, number of binding sites and footprint logo (25,36,37). ATACdb also enables ‘Threshold’ options allowing users to set different thresholds to ensure TFs are high_activity and cell_type-specific for each sample (Figure 2F). In addition, users may click ‘Region ID’ for details about accessible chromatin regions. ATACdb lists the more detailed annotation information including TFs, super-enhancers, typical enhancers, common SNPs, risk SNPs, eQTLs, LD SNPs, DNA methylation sites 3D chromatin interactions and TADs (Figure 2G). The genes associated with accessible chromatin regions are provided through using closest, overlap and proximal identification strategies (47–49) (Figure 2H). The detailed information associated with genes can be displayed, such as gene-disease relationship information and gene expression in different samples from GTEx (58), NCBI (26), ENCODE (20) and CCLE (65) projects. ATACdb also provides the visualization of peak coverage over chromosomes and profiles of peaks binding to the TSS region (Figure 2L).

### A user-friendly interface for browsing accessible chromatin regions

Users can quickly browse samples and customize filters through ‘Biosample type’, ‘Biosample name’, ‘Tissue type’ and ‘Cancer type’ (Figure 2C). The number of records per page can be changed using the ‘Show entries’ drop-down menu. The number statistics of accessible chromatin regions for each sample can be displayed on the page. Importantly, users may further click on the ‘Sample ID’ to view accessible chromatin regions for a given sample.

### Online analysis tools

ATACdb provides two practical analysis tools. One is the ‘Differential-Overlapping-Region’ analysis tool, the other is the ‘Overlapping accessible chromatin regions bound by two TFs’ analysis tool. The ‘Differential-Overlapping-Region’ analysis tool can calculate similarities and differences between accessible chromatin regions of two samples. When users submit two samples of interest, the tool will compare the regions between two samples and extract all regions overlapping at least one base between the two samples. For these overlapping regions, the tool further shows the length of the overlapping regions and overlapping ratio (the ratio of overlapping length to total length). Moreover, we can divide them into four overlapping types. For the non-overlapping regions, we consider them as differential regions, and extract these regions of the two samples respectively. Finally, ATACdb will show these differential and overlapping regions between two samples with their detailed information, including genomic region, region length, region number, overlapping ratio and overlapping type (Figure 2I). The high overlapping ratio indicates more similarity between two accessible chromatin regions. For the ‘Overlapping accessible chromatin regions bound by two TFs’ analysis tool, users can submit two TF names and the window length of TF-binding sites. This tool can calculate overlapping regions based on TF-binding sites. ATACdb will show these overlapping regions with overlapping lengths and overlapping ratios (Figure 2J). This analysis can further help users analyze the overlapping regions bound by two TFs of interest in the accessible chromatin regions.

### Personalized genome browser and data visualization

ATACdb provides a powerful genome browser to help users to intuitively view proximity information of accessible chromatin regions in the genome. We developed a personalized genome browser using JBrowse (66) and added many useful tracks such as accessible chromatin regions, enhancers, super-enhancers, genes, SNPs and TADs (Figure 2M). ATACdb can exhibit chromatin accessibility-associated pie charts of chromosome distribution. In addition, ATACdb provides visualization of TF footprint logos (Figure 2F), histograms of expression of TFs binding to chromatin accessibility regions and the relationships between chromatin accessibility regions and genes (Figure 2H).

### Data download and statistics

Chromatin accessibility regions and the elements of all samples are provided for download in the ‘Download’ page. Users can quickly search and download associated information (Figure 2K). We provided a download of chromatin accessibility region files in ‘.BED’ and ‘.CSV’ format for each sample. For TF footprint analysis, we provided a download of TF footprint files in ‘.txt’ and ‘.pdf ’ format. By clicking ‘pdf’, users can download the corresponding footprint logos in a compressed file. ATACdb supports the packaged download of all accessible chromatin regions and TF footprints analysis result. In the ‘Statistics’ page, ATACdb provides digital and graphical displays about accessible chromatin regions and annotation information for users (Figure 2N). In addition, sample information for super-enhancer and chromatin interactions were provided in ATACdb.

## SYSTEM DESIGN AND IMPLEMENTATION

The ATACdb website runs on a Linux-based Apache Web server 2.4.6 (http://www.apache.org). The database was developed using MySQL 5.7.27 (http://www.mysql.com). PHP 5.6.40 (http://www.php.net) was used for server-side scripting. The ATACdb web interface was built using Bootstrap v3.3.7 (https://v3.bootcss.com) and JQuery v2.1.1 (http://jquery.com). ECharts (http://echarts.baidu.com) was used to be a graphical visualization framework. This database has been tested using Mozilla Firefox, Google Chrome and Internet Explorer web browsers.

ATACdb is freely available to the research community at (http://www.licpathway.net/ATACdb) and requires no registration or login.

## DISCUSSION

Accessible chromatin is closely associated with various biological processes and human diseases, and is coupled with exquisite tissue/cell-specificity. There is an urgent need to comprehensively collect and effectively process human chromatin accessibility data. Some databases, such as GTRD (21), EpiRegio (22) and DeepBlue (23), store chromatin accessibility data based on DNase-seq datasets. However, they focus on gene regulation for ChIP-seq and DNase-seq data, and only provide some chromatin accessibility data. Although OCHROdb (24) stores many chromatin accessibility data, it only supports DNase-I samples (Supplementary Table S1) (Supplementary Material S1). The existing databases, such as Cistrome (18), TCGA (19) and ENCODE (20), store chromatin accessibility data based on ATAC-seq data. However, there is no chromatin accessibility database that focuses on collecting comprehensive chromatin accessibility regions with detailed annotation information and analyses about human ATAC-seq data. ENCODE (20) focuses on gene regulation or histone modification. In ENCODE, the number of human ATAC-seq samples is merely about 50 (20). ATACdb documents a total of 52 078 883 regions from over 1400 chromatin accessibility ATAC-seq samples. There are about 30 times more samples than that in ENCODE. TCGA (19) provides insights into principles of epigenetic regulation limited on ranges of 23 primary human cancers. TCGA only supported cancer-related ATAC-seq samples. ATACdb focuses on providing human chromatin accessibility data in various tissue/cell types. Moreover, the number of samples in ATACdb is about four times than in TCGA (19). Compared to all existing databases such as Cistrome (18), TCGA (19) and ENCODE (20), ATACdb provides two additional useful strategies for inferring TF binding within chromatin accessibility regions including TF footprint analysis and motif scan, as well as quality assurance process by measuring mean insert size. More importantly, ATACdb integrates a large amount of genetic and epigenetic annotation information. Overall, ATACdb is a powerful resource for chromatin accessibility data with the most comprehensive annotation information (Table 1 and Supplementary Table S1).

**Table 1.** Comparison of accessibility information in ATACdb with other databases

Function type	Data type/Specific function	ATACdb	Cistrome	TCGA	ENCODE
Quality control	Mean insert size	✓			
	Standard deviation	✓			
	TSS enrichment score	✓			✓
	Fraction of reads in peaks	✓			✓
	Diagnostic plot ^a^	✓	✓		
TF footprint	Tag Count ^b^	✓			
	TF protection score ^c^	✓			
	Number of binding sites	✓			
	Footprint logo	✓			
Annotation	Strategies of accessible chromatin region associated genes ^d^	3 ^e^	1 ^f^		
	Common SNP	✓			
	Risk SNP	✓			
	eQTL	✓			
	LD SNP	✓			
	Super-enhancer	✓			
	Enhancer	✓			
	Methylation site	✓			
	Chromatin interaction	✓			
	TAD	✓			
Peak annotation visualization	Genomic feature distribution	✓			
	Peak relative to TSS distribution	✓			
Genome browser	Accessible chromatin region	✓	✓		
	SNP	✓			
	Common SNP	✓			
	Risk SNP	✓			
	Super-enhancer	✓	✓		
	Enhancer	✓			
	TFBS conserved	✓			
	TAD	✓			
Analysis functions	Differential-Overlapping-Region analysis ^g^	✓			
	Overlapping accessible chromatin regions bound by two TFs analysis ^h^	✓			
Data browse	Simple information browse	✓	✓	✓	✓
	Browse based on samples classification ^i^	✓			
	Region statistics for each sample	✓			
	Alphanumerically sortable table	✓			

^a^Insert size distribution plot.

^b^Number of reads around TFBSs used to rank footprint predictions.

^c^Footprints with potentially short residence times.

^d^Accessible chromatin region associated genes obtained by different strategies or algorithms.

^e^Closest, overlap and proximal genes were identified by ROSE_geneMapper.

^f^Putative targets were identified by BETA.

^g^Analyze differential and overlapping accessible chromatin regions.

^h^Analyze overlapping accessible chromatin regions bound by two TFs.

^i^Classification of samples including Biosample type, Tissue type, Cancer type and Biosample name.

ATACdb provides a user-friendly interface to query, browse, analyze and visualize chromatin accessibility regions and detailed information about them. We compared ATACdb with other databases for information and functions, which showed the advantages of ATACdb (Table 1 and Supplementary Table S1). These advantages includes (i) QC guidelines for ATAC-seq data that allow users to measure the quality of chromatin accessibility experiments; (ii) the accurate inference of TF binding from DNA sequences using TF footprint analysis; (iii) the comprehensive genetic and epigenetic annotation of chromatin accessibility regions including TFs, super-enhancers, typical enhancers, common SNPs, risk SNPs, eQTLs, LD SNPs, DNA methylation sites 3D chromatin interactions and TADs; (iv) the visualization function to annotate genomic region of peaks; (v) useful and full-featured online analysis tools such as ‘Differential-Overlapping-Region analysis’ and ‘Overlapping accessible chromatin regions bound by two TFs’; (vi) a customized genome browser for intuitively viewing proximity information of accessible chromatin regions and adding a lot of useful tracks; (vii) user-friendly displays accessible chromatin region and associated annotation information with interactive tables.

ATACdb provides a large number of chromatin accessibility regions and comprehensive detail information about standardized curation, QC, TF footprint, and other annotation information. In future versions, ATACdb will follow two main directions. First, we will extend the range of species and further increase annotation information. Second, we will add further practical analysis functions. Overall, ATACdb is by far the most comprehensive platform for curated, annotated and analyzed accessible chromatin data. ATACdb can also help users to understand more potential biological functions in accessible chromatin regions. We extend ATACdb to be useful for both transcriptional and (epi)genetic regulation studies.

## Supplementary Material

gkaa943_Supplemental_Files
