# Supplementary material for: ATACdb: a comprehensive human chromatin accessibility database
Source: Nucleic Acids Res. 2020 Oct 30;49(D1):D55–64. doi: 10.1093/nar/gkaa943 (PMC7779059; doi:10.1093/nar/gkaa943)
Supplement: gkaa943_Supplemental_Files [file gkaa943_supplemental_files.zip › Supplementary Figure 1.pdf]

Supplementary Figure 1

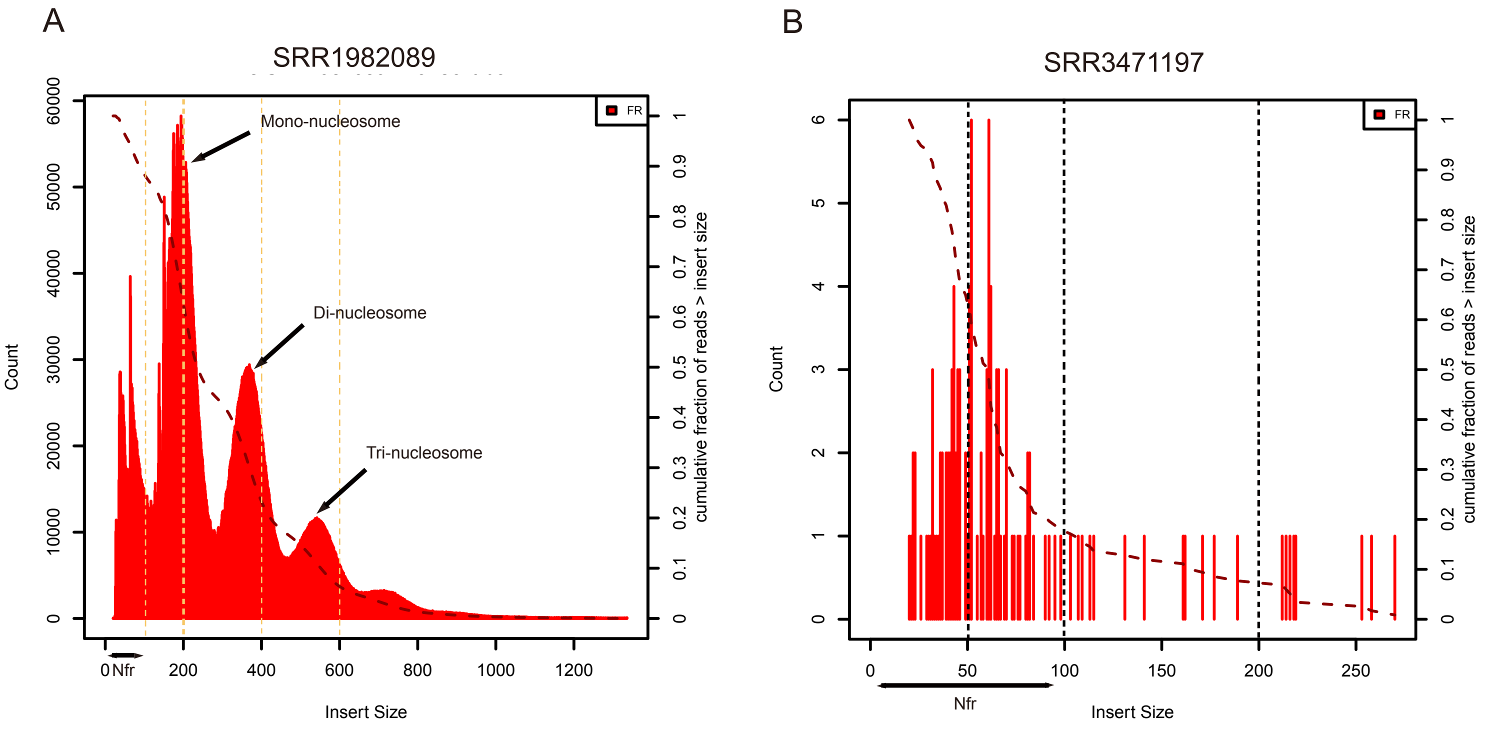

Supplementary Figure 1. QC reports for two representative ATAC-seq datasets: SRR1982089 and SRR3471197. (A) Histogram of the mean insert size distribution showing high-quality ATAC-seq experiment, which displayed decreasing and periodical peaks corresponding to the nucleosome free regions (nfr), mono-, di- and tri-nucleosome. (B) Histogram of mean insert size distribution showing low-quality ATAC-seq experiment.
