# Supplementary material for: ATACdb: a comprehensive human chromatin accessibility database
Source: Nucleic Acids Res. 2020 Oct 30;49(D1):D55–64. doi: 10.1093/nar/gkaa943 (PMC7779059; doi:10.1093/nar/gkaa943)
Supplement: gkaa943_Supplemental_Files [file gkaa943_supplemental_files.zip › Supplementary Table 1.docx]

**Supplementary Table 1.** Comparison of ATACdb with other resources

| Function type | Data type/Specific function | ATACdb | OCHROdb | GTRD | EpiRegio | DeepBlue |
| --- | --- | --- | --- | --- | --- | --- |
| Sample type | ATAC-seq sample | ✔ |  |  |  |  |
| Quality control | Quality control of chromatin accessibility samples | ✔ ^a^ | ✔ ^b^ |  |  |  |
| TF | Footprint analysis | ✔ ^c^ |  | ✔ ^d^ |  |  |
|  | TF binding motif analysis | ✔ ^e^ |  |  | ✔ ^f^ |  |
| Annotation | Strategies of accessible chromatin region associated genes | 3 ^g^ |  |  | 1 ^h^ |  |
|  | Common SNP | ✔ |  |  |  |  |
|  | Risk SNP | ✔ |  |  |  |  |
|  | eQTL | ✔ |  |  |  |  |
|  | LD SNP | ✔ |  |  |  |  |
|  | Super-enhancer | ✔ |  |  |  |  |
|  | Enhancer | ✔ |  |  |  |  |
|  | Methylation | ✔ |  |  |  |  |
|  | Chromatin interaction | ✔ |  |  |  |  |
|  | TAD | ✔ |  |  |  |  |
| Peak annotation  visualization | Genomic feature distribution | ✔ |  |  |  |  |
|  | Peak relative to TSS distribution | ✔ |  |  |  |  |
| Genome browser | Accessible chromatin region | ✔ | ✔ | ✔ | ✔ |  |
|  | SNP | ✔ |  |  |  |  |
|  | Common SNP | ✔ |  |  |  |  |
|  | Risk SNP | ✔ |  |  |  |  |
|  | Super-enhancer | ✔ |  |  |  |  |
|  | Enhancer | ✔ |  |  |  | ✔ |
|  | TFBS | ✔ |  | ✔ |  |  |
|  | TAD | ✔ |  |  |  |  |
| Analysis functions | Differential-Overlapping-Region analysis ^i^ | ✔ |  |  |  |  |
|  | Overlapping accessible chromatin regions bound by two TFs analysis ^j^ | ✔ |  |  |  |  |
| Search options | Search by genomic region | ✔ | ✔ |  | ✔ |  |
|  | Search by tissue type | ✔ |  |  |  |  |
|  | Search by TF | ✔ |  | ✔ |  |  |
|  | Search by gene | ✔ |  |  | ✔ |  |

a. Four quality metrics including mean insert size, standard deviation, TSS enrichment score and FriP.

b. One replication-based quality metric.

c. Footprints with the protection score, TC, number of binding sites and footprint logo.

d. DNase footprints identified by DNase-seq.

e. Motif scan analysis using FIMO.

f. Motif analysis using PASTAA.

g. Closest, overlap and proximal genes were identified by ROSE_geneMapper.

h. Predict gene expression using the STITCHIT algorithm.

i. Analyze differential and overlapping accessible chromatin regions.

j. Analyze overlapping accessible chromatin regions bound by two TFs.
