# Supplementary material for: ATACdb: a comprehensive human chromatin accessibility database
Source: Nucleic Acids Res. 2020 Oct 30;49(D1):D55–64. doi: 10.1093/nar/gkaa943 (PMC7779059; doi:10.1093/nar/gkaa943)
Supplement: gkaa943_Supplemental_Files [file gkaa943_supplemental_files.zip › Supplementary_Material_1.docx]

**Supplementary Material 1. Functions comparison ATACdb with others**

(1). ATACdb documented a total of 52,078,883 chromatin accessibility regions from over 1,400 ATAC-seq samples. GTRD, EpiRegio and DeepBlue supported some chromatin accessibility data, and focused on gene regulation for ChIP-seq and DNase-seq data. Although OCHROdb is a database based on chromatin accessibility data, it only supported DNase-I samples (Supplementary Table 1).

(2). ATACdb provided four QC metrics of ATAC-seq samples, including mean insert size, standard deviation, TSS enrichment score, FRiP and diagnostic plot. GTRD only supported quality control for ChIP-seq data, which do not provide QC of ATAC-seq data. OCHROdb supported one simple QC metrics for DNase-I samples. Others do not support such QC metrics for chromatin accessibility regions (Supplementary Table 1).

(3). ATACdb provided comprehensive (epi)genetic annotation information in chromatin accessibility regions, including SE, TE, TF footprint, motif scan, SNP, eQTLs, DNA methylation sites, 3D chromatin interactions and TADs. Others do not fully provide such annotation information for ATAC-seq data (Supplementary Table 1).

(4). ATACdb is a powerful platform with user-friendly search options to allow users to determine the scope of chromatin accessibility data query through four paths, including ‘Search by genomic region’, ‘Search by tissue type’, ‘Search by TF’ and ‘Search by gene’. OCHROdb only supported query the database by genomic region. Others do not fully support such search options (Supplementary Table 1).

(5). ATACdb supported accurate inference of TF footprint within chromatin accessibility regions. We further calculated three types of assessment score of TF footprint, including TC score, protection score, number of binding sites for each ATAC-seq sample. OCHROdb and DeepBlue do not provide such functionality. EpiRegio only supports enriched motifs, rather than TF footprint analysis. GTRD provided DNase footprints identified by DNase-seq (Supplementary Table 1)

1. . ATACdb provided Chromatin-accessibiliy-region-associated genes through three different identification strategies. EpiRegio supported one strategy to identify distant target genes. Others do not support such functions (Supplementary Table 1).
